# Addressing unsafe abortion in India: A mixed-methods systematic review protocol

**DOI:** 10.1371/journal.pone.0359623

**Published:** 2026-10-27

**Authors:** Pratibha Dhiman, Barsha Gadapani Pathak, Subhanwita Manna, Smiteerekha Sahoo, Vani Kandpal, Bijaya Kumar Mishra, Narayana Swamy D M, Subrata Kumar Palo, Tanveer Rehman, Reema Mukherjee, Sanghamitra Pati

**Affiliations:** 1 Indian Council of Medical Research, New Delhi, India; 2 Society for Applied Studies, New Delhi, India; 3 ICMR-National Institute of Health Research, Bhubaneswar, Odisha, India; MAMTA Health Institute for Mother and Child, INDIA

## Abstract

**Background:**

Unsafe abortion remains a critical public health challenge globally and in India, despite the legal provision under the Medical Termination of Pregnancy (MTP) Act for over 50 years. Multiple barriers -including limited access to trained providers, inequities in health facility readiness, stigma, poor awareness of legal entitlements, and widespread reliance on self-managed medical abortion- contribute to unsafe practices, which result in preventable maternal morbidity and mortality. Evidence on hazardous abortion in India is fragmented across facility-based studies, community surveys, and qualitative research. This protocol outlines methods for a mixed-methods systematic review integrating quantitative and qualitative data to comprehensively assess the prevalence of unsafe abortion, its determinants, associated morbidities, decision-making pathways, and the role of providers, methods, and settings.

**Methods:**

The review will include quantitative, qualitative, and mixed-methods studies conducted in India and published from 1972 onwards. Eligible quantitative designs include surveys, cross-sectional, cohort, case–control, quasi-experimental studies, randomised controlled trials, and hospital series with at least ten instances. Qualitative designs will include studies using interviews, focus groups, ethnography, or participatory approaches. Mixed-methods studies will be eligible if India-specific results can be extracted. The electronic databases to be searched include PubMed/MEDLINE, Embase, Scopus, Web of Science, CINAHL supplemented by grey literature from WHO, UNFPA, the Ministry of Health and Family Welfare, and other national sources. Two reviewers will independently screen studies, extract data, and appraise quality. The Newcastle – Ottawa Scale will be used for observational studies, the Cochrane RoB 2 tool for trials, the Critical Appraisal Skills Programme (CASP) or Joanna Briggs Institute (JBI) checklists for qualitative studies, and the Mixed Methods Appraisal Tool (MMAT) for mixed-methods studies. Quantitative data will be synthesised using random-effects meta-analysis, while qualitative findings will be analysed through thematic synthesis guided by abortion decision-making trajectories. Integration will follow a convergent segregated design.

**Discussion:**

This review will generate an integrated synthesis of unsafe abortion in India, including prevalence, morbidities, determinants, women’s decision-making, and the contextual role of providers and settings. Findings may inform national policy, programmatic interventions, and strategies to strengthen Comprehensive Abortion Care under the National Health Mission. Anticipated challenges include heterogeneity in definitions of “unsafe abortion,” variation in outcome measures, and gaps in subnational data. Despite these limitations, the review will provide essential evidence to guide policy and improve women’s health outcomes.

**Registration:**

This protocol is reported in accordance with PRISMA-2020 guidelines and has been registered with the Open Science Framework (OSF). [https://doi.org/10.17605/OSF.IO/R4JPT].

## Background

Unsafe abortion constitutes a public health challenge both worldwide and in India. The World Health Organization describes unsafe abortion as the termination of an unintended pregnancy performed by individuals who do not have the required medical training, or conducted in settings that fail to meet basic medical and safety standards, or involving both of these conditions [1]. Importantly, unsafe abortion is not a uniform practice but exists along a continuum of risk, influenced by the method used, the provider’s level of skill, and the conditions under which the procedure is performed. To capture this variation in risk, unsafe abortion is classified as ‘less safe’ or ’least safe’ depending on the method used, the provider, and the conditions under which the procedure is performed [20].

Globally, around 45% of the nearly 73 million induced abortions that take place each year occur in unsafe circumstances, accounting for a considerable share of maternal ill-health and an estimated 4.7–13.2% of maternal deaths worldwide [2, 3]. Regions with restrictive access see the highest unsafe-abortion mortality rate. In such settings, complications like incomplete abortion, severe haemorrhage, infection/sepsis, uterine perforation, and internal organ injury are common [4].

In this context, India presents a unique paradox of having a long-standing legal framework for abortion alongside a persistent burden of unsafe procedures. India legalised abortion in 1971 through the Medical Termination of Pregnancy (MTP) Act, positioning itself among the earliest developing countries to establish a legal framework for abortion [5]. The legislative framework for abortion in India has evolved. The original MTP Act allowed abortion under conditions such as risk to the life of the woman, severe injury to her physical or mental health, pregnancy resulting from rape, and substantial risk of fetal congenital abnormalities [5, 6]. Amendments in 2002 expanded the scope of medical abortion by allowing the use of mifepristone and misoprostol and broadened the base of providers to include trained practitioners [5,7]. More recently, the 2021 amendment extended the gestational limit for specific categories of women to 24 weeks and recognised the rights of survivors of rape, minors, and women with significant fetal anomalies [5,8]. Despite progressive legislation, many women in India still lack access to safe, legal abortion services due to implementation gaps, limited-service availability, and pervasive stigma [8,9].

In 2018, Singh and co-authors reported that approximately 15.6 million abortions took place in India in 2015. Their analysis indicated that about one-fifth (22%) of these procedures were carried out in health facilities, while the majority (73%) were medication abortions obtained and used outside formal health settings, and a small proportion involved non-medication methods used outside health facilities [10]. Another study covering nine Indian states (2007–2011) classified 67.1% of reported abortions as unsafe [11]. The United Nations Population Fund reports that unsafe abortion ranks as the third most common cause of maternal mortality in India, resulting in roughly eight maternal deaths each day [12]. Hospital-based studies corroborate that complications of unsafe abortion (e.g., haemorrhage, sepsis, uterine injury) are among the major contributors to maternal morbidity and mortality in many regions of India [13].

Inequities in access further complicate the issue. Rural women, adolescents, unmarried women, and those from marginalised caste or tribal communities face multiple barriers, including stigma, lack of confidentiality, and limited availability of facilities [13,14]. Even where services exist, women often encounter judgmental attitudes from providers, requirements for spousal or parental consent despite legal autonomy, and the threat of breaches of confidentiality [14,15]. For many, the perceived risks of stigma or social exposure outweigh the health risks, driving them toward clandestine or unsafe methods [15].

Research shows that unsafe abortion in India causes complications such as incomplete abortion, haemorrhage, sepsis, uterine perforation, organ injury, and maternal death [4]. These often result from delayed care, unsafe methods, or untrained providers, while morbidity estimates vary due to inconsistent definitions and reliance on facility-based data [16,17]. Determinants extend beyond clinical gaps to socio-cultural and economic factors, including poverty, gender inequality, lack of contraception, stigma, and poor knowledge of legal rights [18]. Family members, especially male partners, strongly influence decisions, sometimes coercively [18]. Women often prioritise secrecy and confidentiality over health, perceiving “safety” in social, legal, and reputational terms rather than biomedical risk [15,19]. Provider attitudes play a key role: judgmental responses deter formal care, while supportive providers, telemedicine, and hotlines expand safer options [15,19].

There is a need for a comprehensive, India-specific synthesis that brings together data on prevalence, clinical outcomes, determinants, and women lived experiences. Existing global reviews include India but lack a focused analysis [5,11,14]. This review will address that gap through a mixed-methods approach, integrating quantitative estimates with qualitative insights to generate evidence directly relevant to Indian policy and programs.

## Aim

To develop an integrated and thorough summary of existing evidence on unsafe abortion in India by conducting a mixed-methods systematic review.

## Objectives

- Estimate the prevalence of unsafe abortion at the national and regional levels of India.
- Estimate the prevalence of unsafe abortion-related morbidities and complications.
- Identify determinants of unsafe abortion at personal, societal, and health system levels.
- Synthesise qualitative insights into women’s decision-making and care-seeking pathways.

## Methodology

This review will be conducted in accordance with the Preferred Reporting Items for Systematic Review and Meta-Analysis Protocols (PRISMA-2020) guidelines [21].A mixed-methods approach will be used to combine and integrate both quantitative and qualitative evidence.

### Protocol registration

The protocol is registered with Open Science Framework (OSF) [https://doi.org/10.17605/OSF.IO/R4JPT]. Any amendments during the review process will be documented and updated in the OSF record.

### Ethics approval and consent to participate

Not applicable. This review will synthesise data from published and publicly available sources.

### Eligibility criteria

This review will focus on studies conducted in India that examine unsafe abortion among women of reproductive age (15–49 years). A wide range of quantitative study designs will be included, such as cross-sectional, cohort, case–control, quasi-experimental studies, and randomized controlled trials. Qualitative studies employing methods like in-depth interviews, focus group discussions, ethnographic approaches, or participatory research will also be considered. Mixed-methods studies conducted across multiple countries will be eligible provided that India-specific results are reported separately.

To capture evidence generated following the enactment of the Medical Termination of Pregnancy (MTP) Act as a major policy milestone, only studies published from January 1972 onward will be included. Studies will be excluded if they do not present findings specific to India, are based solely on modelling without primary data, consist of small case reports, or address only spontaneous abortion without clearly distinguishing it from induced abortion.

### Information sources and search strategy

A detailed search strategy will be designed in collaboration with a research librarian with expertise in conducting systematic reviews. Searches will be carried out across major electronic bibliographic databases, including PubMed/MEDLINE, Embase, Scopus, Web of Science Core Collection, CINAHL. To ensure adequate coverage of India-focused research that may not be indexed in international databases, additional searches will be conducted in IndMED, MedIND, and J-Gate.

Grey literature will also be explored by reviewing institutional repositories, government sources such as the Ministry of Health and Family Welfare (MoHFW) and the National Health Mission (NHM), and national data systems including the Health Management Information System (HMIS) and the Sample Registration System (SRS). Furthermore, reports and publications from international organisations such as the World Health Organization (WHO) and the United Nations Population Fund (UNFPA), along with documents from Indian non-governmental organisations working in reproductive health, will be examined.

The search will use a combination of Medical Subject Headings (MeSH) and relevant keywords related to abortion, safety, and the Indian context. Separate search strategies will be developed for quantitative and qualitative studies, with The detailed search strategies for quantitative and qualitative studies are provided in S1 File and S2 File, respectively.

This will be adapted for other databases using their specific indexing terms. The search strategy will be peer-reviewed by another expert. All searches will be documented, and results will be exported to citation manager software for de-duplication [22].

### Study selection

All titles and abstracts identified through the search will be independently assessed by two reviewers using predefined inclusion and exclusion criteria. Articles that meet, or appear likely to meet, these criteria will then be evaluated through a full-text review [23]. At each stage of the review process, any differences in judgment will be addressed through discussion. If agreement cannot be achieved, a senior third reviewer will make the final decision. The reasons for excluding studies at the full-text screening stage will be documented [23]. The study selection process will be reported using the PRISMA 2020 flow diagram [21]. The completed PRISMA 2020 checklist is provided in S3 Checklist.

### Data extraction

Data extraction will be carried out independently by two reviewers using a standardized data collection form. Information to be extracted will cover bibliographic details (such as authors, year of publication, journal, and funding source), study features (including design, setting, study period, sample size, and participant characteristics), and outcomes related to unsafe abortion.

For quantitative studies, extracted data will include estimates of prevalence or incidence, details on abortion-related morbidities and complications (including type and severity), associated determinants with corresponding effect sizes and confidence intervals, and information on service providers, methods used, and settings of care. For qualitative studies, data extraction will focus on study context, participant profiles, and key themes concerning women’s decision-making, stigma, autonomy, and pathways to care. Mixed-methods studies will be reviewed and coded to capture both quantitative and qualitative components.

Where critical information is missing, attempts will be made to contact the original study authors. All extracted data will be cross verified between reviewers, and any inconsistencies will be resolved through discussion and agreement.

### Status of the review and timeline

This manuscript reports a protocol for a mixed-methods systematic review. At the time of submission, literature searches, study screening, data extraction, and data synthesis have not yet commenced. The search strategy has been finalised, with database searches planned by February 2026.

## Operational definition

**Abortion:** defined by the WHO as ending a pregnancy before 20 weeks of gestation or if the baby weighs less than 500 grams [24].

***Unsafe abortion:*** procedure for terminating an unintended pregnancy carried out by individuals lacking the necessary skills, in an environment that does not meet minimal medical standards, or both [24–26].

Prevalence of unsafe abortion: The proportion of women of reproductive age who have ever had an unsafe abortion (lifetime prevalence) or within a defined period (e.g., last 24 months).

**Incidence of unsafe abortion:** The number of unsafe abortions that occur in a population during a specified time period, usually expressed per 1,000 women of reproductive age (15–49 years) per year, or per 100 live births [45].

**Reproductive age:** Women under the age group of 15–49 years or the period of reproduction [43].

**Less safe abortions: “**Abortions are less safe if only one of the two criteria were met, either the abortion was done by a trained provider but with an outdated method (e.g., sharp curettage) or a safe method of abortion (e.g., misoprostol) was used but without adequate information or support from a trained individual [44].

**Least safe abortions: “**Abortions are least safe if they are provided by untrained individuals using dangerous methods, such as ingestion of caustic substances, insertion of foreign bodies, or use of traditional concoctions”[44].

### Outcomes

The main outcomes of interest are the magnitude of unsafe abortion in India, measured through its prevalence and incidence, along with the prevalence and incidence of complications arising from unsafe procedures. These complications encompass conditions such as excessive bleeding, infection, injury to the uterus or genital tract, damage to the bowel or bladder, kidney failure, anaemia, near-miss events, and deaths attributable to unsafe abortion.

Secondary outcomes will explore the factors associated with unsafe abortion, including socio demographic characteristics, interpersonal relationships, and broader structural or health system influences. The review will also assess women’s decision-making pathways and document the types of providers involved, the methods used, and the settings in which unsafe abortions occur.

### Data synthesis and analysis

For the quantitative data, prevalence and morbidity estimates will be combined using a random-effects modelling approach [27].Transformations such as Freeman–Tukey or logit will be applied to stabilise variance [28]. Heterogeneity among studies will be evaluated using I^2^ and τ^2^ statistics and, where possible, further examined through subgroup analyses and meta regression [29]. Forest plots will illustrate pooled and individual study estimates. For determinants of unsafe abortion, adjusted odds ratios or relative risks or events will be pooled if definitions and exposures are sufficiently consistent; otherwise, results will be synthesised narratively.

To provide qualitative evidence, a thematic synthesis will be conducted. The qualitative analysis will include coding the data, creating descriptive themes, and deriving analytical themes. While the nine decision-making trajectories from prior global reviews will provide a guiding framework, they will not limit the emergence of new, inductively derived themes.

Mixed-methods integration will be undertaken using a convergent-segregated approach, whereby quantitative and qualitative findings are synthesised separately and then integrated through joint displays. This will allow us to compare, contrast, and enrich quantitative trends (such as the proportion of women using self-managed medical abortion) with qualitative insights (such as women’s motivations for secrecy or preference for certain providers)

### Assessment of bias and study quality

The risk of bias will be evaluated using tools suited to the specific study design. Observational studies, including cross-sectional, cohort, and case–control studies, will be appraised using the Newcastle–Ottawa Scale (NOS) [30]. Randomized controlled trials, if included, will be evaluated using the Cochrane Risk of Bias 2 (RoB 2) tool. with visualisations generated using robvis or RevMan software [31]. Qualitative studies will be appraised using either the Joanna Briggs Institute(JBI) qualitative assessment tool, [32] depending on the study type. Mixed-methods studies will be assessed using the Mixed Methods Appraisal Tool (MMAT 2018/2022) [33].

Two reviewers will independently assess each study, and discrepancies will be resolved through consensus. Studies will not be excluded solely because of their risk of bias, however, the results of the quality assessment will guide the synthesis and will be reported transparently.

### Subgroup analyses

Subgroup analyses will be conducted to explore variations in unsafe abortion prevalence and outcomes. These will include subnational differences by state and region, rural versus urban populations, age groups, marital status, caste or tribal affiliation, and socio-economic groups. Provider-related subgroups (skilled versus unskilled, formal versus informal), method-related subgroups (surgical versus medical, self-managed versus facility-based), and setting-related subgroups (home, primary, secondary, tertiary facilities) will also be analysed. Policy era subgroups will be defined as 1972–2002 (era before the legalised the use of oral misoprostol and mifepristone for abortion), 2002–2021 (era before MTP Amendment act), and post-2021 (after the most recent MTP amendment).

### Certainty in evidence

The certainty of quantitative evidence will be evaluated using the GRADE (Grading of Recommendations, Assessment, Development and Evaluation) approach, taking into account factors such as risk of bias, inconsistency, indirectness, imprecision, and potential publication bias. For qualitative evidence, confidence in the findings will be assessed using the GRADE-CERQual approach [34]. The Summary of Findings tables will display the key results together with the corresponding certainty ratings.

## Discussion

Unsafe abortion is not just a statistic in India; it is a lived reality shaped by daily choices, social pressures, and persistent gaps in the health system [10,15,16]. Although legal provisions exist, countless women still cannot access safe services for reasons that go far beyond policy alone. Stigma, lack of privacy, judgment from providers, and practical constraints push many toward secrecy or unsafe options, especially in rural areas and among marginalised groups [11,15,18].

The importance of this review lies in its integration of diverse types of evidence. Quantitative data will provide national and sub-national prevalence estimates and morbidity profiles, while qualitative data will illuminate the social and cultural dynamics that drive women’s choices [35–37]. By integrating both, the review will address not just how many women experience unsafe abortion, but why they do, and under what circumstances. This combination is particularly valuable in India, where abortion is both legally available and highly stigmatised, and where disparities in access are shaped by geography, socio-economic status, and gender norms [16,38,39].

The findings are expected to have implications for multiple stakeholders. For policymakers, the review will highlight gaps in service coverage and inform implementation of Comprehensive Abortion Care guidelines [7] under the National Health Mission. For clinicians and public health practitioners, insights into morbidity profiles will guide preparedness for managing complications [42]. For advocacy groups, qualitative findings on decision-making and stigma will provide evidence to support interventions that strengthen women’s autonomy and confidentiality [18].

Several limitations are anticipated. First, heterogeneity in how studies define and measure unsafe abortion may constrain the ability to pool results. We will attempt to address this by mapping studies to the WHO framework of method, provider, and setting [1]. Second, facility-based studies may over-represent severe cases, while community surveys may under-report abortion because of stigma [6,18]. Triangulation of multiple data sources will be used to mitigate these biases. Third, subnational analyses may be limited by data availability, with some states underrepresented [16, 40]. Even so, highlighting these gaps will itself be valuable for guiding future research.

Ultimately, this review aims not only to document the burden of unsafe abortion but also to capture the lived realities of women navigating abortion decision-making in India. By combining epidemiological data with narratives of choice, constraint, and negotiation, the review will provide evidence that is both scientifically rigorous and deeply relevant to women’s lives [18,41].

## Supporting information

S1 File Search strategy for quantitative studies. (DOCX)

S2 File Search strategy for qualitative studies. (DOCX)

S3 Checklist Completed PRISMA 2020 checklist. (DOCX)

## Abbreviations

MTP: Medical Termination of Pregnancy
MoHFW: Ministry of Health and Family Welfare
NHM: National Health Mission
HMIS: Health Management Information System
PRISMA-2020: Preferred Reporting Items for Systematic Review and Meta-Analysis Protocols
GRADE: Grading of Recommendations Assessment, Development and Evaluation
CERQual: Confidence in the Evidence from Reviews of Qualitative research
NOS: Newcastle–Ottawa Scale
MMAT: Mixed Methods Appraisal Tool
JBI: Joanna Briggs Institute
OSF: Open science framework
