# Supplementary material for: Addressing unsafe abortion in India: A mixed-methods systematic review protocol
Source: PLoS One. 2026 Oct 27;21(10):e0359623. doi: 10.1371/journal.pone.0359623 (PMC13712606; doi:10.1371/journal.pone.0359623)
Supplement: Supplementary file 1 [file pone.0359623.s001.docx]

**Search strategy unsafe abortion (Quantitative studies)**

|  | **Pubmed** | **WOS** | **Embase (via Ovid)** | | **CINAHL (via EBSCO)** | **Scopus** |
| --- | --- | --- | --- | --- | --- | --- |
| **#1** | ("unsafe abortion"[MeSH Terms] OR "unsafe abortion"[Title/Abstract] OR "abortion, induced"[MeSH Terms] OR "abortion"[Title/Abstract] OR "abortion practices"[Title/Abstract] OR "abortion methods"[Title/Abstract] OR "abortion decision"[Title/Abstract]) OR "unsafe MTP*"[Title/Abstract] OR "miscarriage*"[Title/Abstract] OR "unsafe termination of pregnancy*"[Title/Abstract] | ("unsafe abortion" OR "unsafe abortion" OR "abortion, induced" OR abortion OR "abortion practices" OR "abortion methods" OR "abortion decision") OR "unsafe MTP*" OR miscarriage* OR "unsafe termination of pregnancy*" | 'unsafe abortion'/exp OR 'unsafe abortion':ti,ab,kw OR 'abortion induced'/exp OR abortion:ti,ab,kw OR 'abortion practices':ti,ab,kw OR 'abortion methods':ti,ab,kw OR 'abortion decision':ti,ab,kw OR 'unsafe mtp*':ti,ab,kw OR miscarriage*:ti,ab,kw OR 'unsafe termination of pregnancy*':ti,ab,kw | (MH "Abortion, Induced") OR (MH "Abortion, Therapeutic") OR (MH "Abortion, Legal") OR (MH "Abortion, Criminal") OR (MH "Abortion") OR ("unsafe abortion" OR abortion OR "abortion practices" OR "abortion methods" OR "abortion decision" OR "unsafe termination of pregnancy" OR miscarriage*) | | ("unsafe abortion" OR  "unsafe abortion" OR "abortion, induced"   OR "abortion"   OR "abortion practices"   OR "abortion methods"OR  "abortion decision") OR  "unsafe MTP*"OR   "miscarriage*"OR "unsafe termination of pregnancy*" |
| **#2** | ("quantitative"[Title/Abstract] OR "cross-sectional"[Title/Abstract] OR "survey"[Title/Abstract] OR "cohort"[Title/Abstract] OR "case-control"[Title/Abstract] OR "randomized controlled trial"[Publication Type] OR "prevalence"[Title/Abstract] OR "incidence"[Title/Abstract] OR "quasi-experimental"[Title/Abstract] OR "survey data"[Title/Abstract] OR "quantitative analysis"[Title/Abstract] OR "secondary data analysis"[Title/Abstract] OR "record-based"[Title/Abstract] OR "register-based"[Title/Abstract] OR "facility-based"[Title/Abstract] OR "data analysis"[Title/Abstract] OR "burden*"[Title/Abstract] OR "numerical data"[Title/Abstract] OR "routine data"[Title/Abstract] OR "longitudinal"[Title/Abstract] OR "retrospective"[Title/Abstract] OR "prospective"[Title/Abstract] OR "descriptive"[Title/Abstract] OR "statistical analysis"[Title/Abstract] OR "epidemiologic studies"[MeSHTerms] ) | (quantitative OR cross-sectional OR survey OR cohort OR case-control OR "randomized controlled trial[Publication Type]" OR prevalence OR incidence OR quasi-experimental OR "survey data" OR "quantitative analysis" OR "secondary data analysis" OR record-based OR register-based OR facility-based  OR "data analysis" OR burden* OR "numerical data" OR "routine data" OR longitudinal OR retrospective OR prospective OR descriptive OR "statistical analysis" OR "epidemiologic studies[MeSHTerms]") | quantitative:ti,ab,kw OR 'cross-sectional':ti,ab,kw OR survey:ti,ab,kw OR cohort:ti,ab,kw OR 'case-control':ti,ab,kw OR prevalence:ti,ab,kw OR incidence:ti,ab,kw OR 'quasi-experimental':ti,ab,kw OR 'secondary data analysis':ti,ab,kw OR 'record based':ti,ab,kw OR 'register based':ti,ab,kw  OR 'facility based':ti,ab,kw OR 'data analysis':ti,ab,kw OR burden*:ti,ab,kw OR 'routine data':ti,ab,kw OR longitudinal:ti,ab,kw OR retrospective:ti,ab,kw OR prospective:ti,ab,kw OR descriptive:ti,ab,kw OR 'statistical analysis':ti,ab,kw | (MH "Quantitative Studies") OR (MH "Epidemiologic Research") OR (MH "Cohort Studies") OR (MH "Case Control Studies") OR (MH "Cross-Sectional Studies") OR (MH "Prevalence") OR (MH "Incidence") OR (MH "Retrospective Studies") OR (MH "Prospective Studies") OR (MH "Longitudinal Studies") OR  (quantitative OR survey OR cohort OR "case-control" OR prevalence OR incidence OR "cross-sectional" OR longitudinal OR retrospective OR prospective OR descriptive OR "quasi-experimental") | | (TITLE-ABS("quantitative") OR TITLE-ABS("cross-sectional") OR TITLE-ABS("survey") OR TITLE-ABS("cohort") OR TITLE-ABS("case-control") OR "randomized controlled trial[Publication Type]" OR TITLE-ABS("prevalence") OR TITLE-ABS("incidence") OR TITLE-ABS("quasi-experimental") OR TITLE-ABS("survey data") OR TITLE-ABS("quantitative analysis") OR TITLE-ABS("secondary data analysis") OR TITLE-ABS("record-based") OR TITLE-ABS("register-based") OR TITLE-ABS("facility-based") OR TITLE-ABS("data analysis") OR TITLE-ABS("burden*") OR TITLE-ABS("numerical data") OR TITLE-ABS("routine data") OR TITLE-ABS("longitudinal") OR TITLE-ABS("retrospective") OR TITLE-ABS("prospective") OR TITLE-ABS("descriptive") OR TITLE-ABS("statistical analysis") OR "epidemiologic studies[MeSHTerms]") |
| **#3** | ("India"[MeSH Terms] OR "India"[Title/Abstract]) | (India OR India) | 'india'/exp OR india:ti,ab,kw | (MH "India") OR India | | ((MH "India+") OR (TI India OR AB India)) |
|  | #1 AND #2 AND #3 | #1 AND #2 AND #3 | #1 AND #2 AND #3 | #1 AND #2 AND #3 | | #1 AND #2 AND #3 |
| **4** | ("unsafe abortion"[Mesh] OR "unsafe abortion"[tiab] OR "abortion, induced"[Mesh] OR abortion[tiab] OR "abortion practices"[tiab] OR "abortion methods"[tiab] OR "abortion decision"[tiab]) OR "unsafe mtp*"[tiab] OR miscarriage*[tiab] OR "unsafe termination of pregnancy*"[tiab] AND (quantitative[tiab] OR cross-sectional[tiab] OR survey[tiab] OR cohort[tiab] OR case-control[tiab] OR "randomized controlled trial[Publication Type]" OR prevalence[tiab] OR incidence[tiab] OR quasi-experimental[tiab] OR "survey data"[tiab] OR "quantitative analysis"[tiab] OR "secondary data analysis"[tiab] OR record-based[tiab] OR register-based[tiab] OR facility-based[tiab] OR "data analysis"[tiab] OR burden*[tiab] OR "numerical data"[tiab] OR "routine data"[tiab] OR longitudinal[tiab] OR retrospective[tiab] OR prospective[tiab] OR descriptive[tiab] OR "statistical analysis"[tiab] OR "epidemiologic studies[MeSHTerms]") AND (India[Mesh] OR India[tiab]) | ("unsafe abortion" OR "unsafe abortion" OR "abortion, induced" OR abortion OR "abortion practices" OR "abortion methods" OR "abortion decision") OR "unsafe mtp*" OR miscarriage* OR "unsafe termination of pregnancy*" AND (quantitative OR cross-sectional OR survey OR cohort OR case-control OR "randomized controlled trial[Publication Type]" OR prevalence OR incidence OR quasi-experimental OR "survey data" OR "quantitative analysis" OR "secondary data analysis" OR record-based OR register-based OR facility-based OR "data analysis" OR burden* OR "numerical data" OR "routine data" OR longitudinal OR retrospective OR prospective OR descriptive OR "statistical analysis" OR "epidemiologic studies[MeSHTerms]")  AND (India OR India)  ((TS=( "unsafe abortion" OR "induced abortion" OR abortion OR "abortion practices" OR "abortion methods" OR "abortion decision" OR "unsafe mtp*" OR miscarriage* OR "unsafe termination of pregnancy*" )) AND TS=(quantitative OR cross-sectional OR survey OR cohort OR case-control OR prevalence OR incidence OR quasi-experimental OR "survey data" OR "quantitative analysis" OR "secondary data analysis" OR record-based OR register-based OR facility-based OR "data analysis" OR burden* OR "numerical data" OR "routine data" OR longitudinal OR retrospective OR prospective OR descriptive OR "statistical analysis" OR epidemiologic*)) AND TS=(India) | 'unsafe abortion'/exp OR 'unsafe abortion':ti,ab,kw OR 'abortion induced'/exp OR abortion:ti,ab,kw OR 'abortion practices':ti,ab,kw OR 'abortion methods':ti,ab,kw OR 'abortion decision':ti,ab,kw OR 'unsafe mtp*':ti,ab,kw OR miscarriage*:ti,ab,kw OR 'unsafe termination of pregnancy*':ti,ab,kw AND quantitative:ti,ab,kw OR 'cross-sectional':ti,ab,kw OR survey:ti,ab,kw OR cohort:ti,ab,kw OR 'case-control':ti,ab,kw OR prevalence:ti,ab,kw OR incidence:ti,ab,kw OR 'quasi-experimental':ti,ab,kw OR 'secondary data analysis':ti,ab,kw OR 'record based':ti,ab,kw OR 'register based':ti,ab,kw OR 'facility based':ti,ab,kw OR 'data analysis':ti,ab,kw OR burden*:ti,ab,kw OR 'routine data':ti,ab,kw OR longitudinal:ti,ab,kw OR retrospective:ti,ab,kw OR prospective:ti,ab,kw OR descriptive:ti,ab,kw OR 'statistical analysis':ti,ab,kw' AND india'/exp OR india:ti,ab,kw | (MH "Abortion, Induced") OR (MH "Abortion, Therapeutic") OR (MH "Abortion, Legal") OR (MH "Abortion, Criminal") OR (MH "Abortion") OR ("unsafe abortion" OR abortion OR "abortion practices" OR "abortion methods" OR "abortion decision" OR "unsafe termination of pregnancy" OR miscarriage*) AND (MH "Quantitative Studies") OR (MH "Epidemiologic Research") OR (MH "Cohort Studies") OR (MH "Case Control Studies") OR (MH "Cross-Sectional Studies") OR (MH "Prevalence") OR (MH "Incidence") OR (MH "Retrospective Studies") OR (MH "Prospective Studies") OR (MH "Longitudinal Studies") OR  (quantitative OR survey OR cohort OR "case-control" OR prevalence OR incidence OR "cross-sectional" OR longitudinal OR retrospective OR prospective OR descriptive OR "quasi-experimental")  AND (MH "India") OR India | | (INDEXTERMS("unsafe abortion") OR TITLE-ABS("unsafe abortion") OR INDEXTERMS("abortion, induced") OR TITLE-ABS("abortion") OR TITLE-ABS("abortion practices") OR TITLE-ABS("abortion methods") OR TITLE-ABS("abortion decision")) OR TITLE-ABS("unsafe mtp*") OR TITLE-ABS("miscarriage*") OR TITLE-ABS("unsafe termination of pregnancy*") AND (TITLE-ABS("quantitative") OR TITLE-ABS("cross-sectional") OR TITLE-ABS("survey") OR TITLE-ABS("cohort") OR TITLE-ABS("case-control") OR "randomized controlled trial[Publication Type]" OR TITLE-ABS("prevalence") OR TITLE-ABS("incidence") OR TITLE-ABS("quasi-experimental") OR TITLE-ABS("survey data") OR TITLE-ABS("quantitative analysis") OR TITLE-ABS("secondary data analysis") OR TITLE-ABS("record-based") OR TITLE-ABS("register-based") OR TITLE-ABS("facility-based") OR TITLE-ABS("data analysis") OR TITLE-ABS("burden*") OR TITLE-ABS("numerical data") OR TITLE-ABS("routine data") OR TITLE-ABS("longitudinal") OR TITLE-ABS("retrospective") OR TITLE-ABS("prospective") OR TITLE-ABS("descriptive") OR TITLE-ABS("statistical analysis") OR "epidemiologic studies[MeSHTerms]") AND (INDEXTERMS("India") OR TITLE-ABS("India")) |
| 5 | Total studies: 681 | Tota: 727 (date: 4March2026) | 915 | 270 | |  |

WHO Global Index Medicus (GIM)

(("unsafe abortion" OR "induced abortion" OR abortion OR "abortion practices" OR "abortion methods" OR "abortion decision" OR "unsafe mtp*" OR miscarriage* OR "unsafe termination of pregnancy*")) AND ((quantitative OR cross-sectional OR survey OR cohort OR case-control OR prevalence OR incidence OR quasi-experimental OR "survey data" OR "quantitative analysis" OR "secondary data analysis" OR record-based OR register-based OR facility-based OR "data analysis" OR burden* OR "numerical data" OR "routine data" OR longitudinal OR retrospective OR prospective OR descriptive OR "statistical analysis" OR epidemiologic*)) AND ((India OR Indian))

Total: 523 (dated 4^th^ March 2026)
