# Supplementary material for: Addressing unsafe abortion in India: A mixed-methods systematic review protocol
Source: PLoS One. 2026 Oct 27;21(10):e0359623. doi: 10.1371/journal.pone.0359623 (PMC13712606; doi:10.1371/journal.pone.0359623)
Supplement: Supplementary file 2 [file pone.0359623.s002.docx]

**Search strategy unsafe abortion (Qualitative studies)**

|  | **Pubmed** | **WOS** | **Embase (via Ovid)** | **CINAHL (via EBSCO)** | **Scopus** |
| --- | --- | --- | --- | --- | --- |
| **1** | ("unsafe abortion"[Mesh] OR "unsafe abortion"[tiab] OR "abortion, induced"[Mesh] OR abortion[tiab] OR "abortion practices"[tiab] OR "abortion methods"[tiab] OR "abortion decision"[tiab]) "OR "unsafe MTP*""[tiab] OR miscarriage*[tiab] OR "unsafe termination of pregnancy*"[tiab] | ("unsafe abortion" OR "unsafe abortion" OR "abortion, induced" OR abortion OR "abortion practices" OR "abortion methods" OR "abortion decision") "OR "unsafe MTP*"" OR miscarriage* OR "unsafe termination of pregnancy*" | ('unsafe abortion'/exp OR 'unsafe abortion':ti,ab,kw OR 'abortion induced'/exp OR abortion:ti,ab,kw OR 'abortion practices':ti,ab,kw OR 'abortion methods':ti,ab,kw OR 'abortion decision':ti,ab,kw OR 'unsafe mtp*':ti,ab,kw OR miscarriage*:ti,ab,kw OR 'unsafe termination of pregnancy*':ti,ab,kw) | (MH "Abortion, Induced") OR (MH "Abortion, Therapeutic") OR (MH "Abortion, Legal") OR (MH "Abortion, Criminal") OR (MH "Abortion") OR ("unsafe abortion" OR abortion OR "abortion practices" OR "abortion methods" OR "abortion decision" OR "unsafe termination of pregnancy" OR miscarriage*) | (INDEXTERMS("unsafe abortion") OR TITLE-ABS("unsafe abortion") OR INDEXTERMS("abortion, induced") OR TITLE-ABS("abortion") OR TITLE-ABS("abortion practices") OR TITLE-ABS("abortion methods") OR TITLE-ABS("abortion decision")) TITLE-ABS("OR "unsafe MTP*"") OR TITLE-ABS("miscarriage*") OR TITLE-ABS("unsafe termination of pregnancy*") |
| **2** | ("qualitative research"[Mesh] OR qualitative[tiab] OR interview[tiab] OR "focus group"[tiab] OR exploratory[tiab] OR experiences[tiab] OR perception*[tiab] OR beliefs[tiab] OR barriers[tiab] OR narrative*[tiab] OR "thematic analysis"[tiab] OR "content analysis"[tiab] OR "grounded theory"[tiab] OR phenomenology*[tiab] OR "life history"[tiab] OR "participant observation"[tiab] OR open-ended[tiab] OR "unstructured interview"[tiab] OR interpretive[tiab] OR "lived experience*"[tiab] OR "case studies"[tiab] OR ethnography*[tiab] OR in-depth*[tiab] OR semi-structured[tiab]) | ("qualitative research" OR qualitative OR interview OR "focus group" OR exploratory OR experiences OR perception* OR beliefs OR barriers OR narrative* OR "thematic analysis" OR "content analysis" OR "grounded theory" OR phenomenology* OR "life history" OR "participant observation" OR open-ended OR  "unstructured interview" OR interpretive OR "lived experience*" OR "case studies" OR ethnography* OR in-depth* OR semi-structured) | ('qualitative research'/exp OR qualitative:ti,ab,kw OR interview*:ti,ab,kw OR 'focus group*':ti,ab,kw OR perception*:ti,ab,kw OR experience*:ti,ab,kw OR 'in-depth':ti,ab,kw OR 'semi-structured':ti,ab,kw OR 'case stud*':ti,ab,kw OR ethnograph*:ti,ab,kw OR 'grounded theory':ti,ab,kw OR  phenomenolog*:ti,ab,kw OR 'thematic analysis':ti,ab,kw OR 'content analysis':ti,ab,kw) | (MH "Qualitative Research+") OR (TI qualitative OR AB qualitative) OR (TI interview* OR AB interview*) OR (TI "focus group*" OR AB "focus group*") OR (TI exploratory OR AB exploratory) OR (TI experiences OR AB experiences) OR (TI perception* OR AB perception*) OR (TI beliefs OR AB beliefs) OR (TI barriers OR AB barriers) OR (TI narrative* OR AB narrative*) OR (TI "thematic analysis" OR AB "thematic analysis") OR (TI "content analysis" OR AB "content analysis") OR (TI "grounded theory" OR AB "grounded theory") OR (TI phenomenology* OR AB phenomenology*) OR (TI "life history" OR AB "life history") OR (TI "participant observation" OR AB "participant observation") OR (TI open-ended OR AB open-ended) OR (TI "unstructured interview*" OR AB "unstructured interview*") OR (TI interpretive OR AB interpretive) OR (TI "lived experience*" OR AB "lived experience*") OR (TI "case stud*" OR AB "case stud*") OR (TI ethnography* OR AB ethnography*) OR (TI in-depth* OR AB in-depth*) OR (TI semi-structured OR AB semi-structured) | (INDEXTERMS("qualitative research") OR TITLE-ABS("qualitative") OR TITLE-ABS("interview") OR TITLE-ABS("focus group") OR TITLE-ABS("exploratory") OR TITLE-ABS("experiences") OR TITLE-ABS("perception*") OR TITLE-ABS("beliefs") OR TITLE-ABS("barriers") OR TITLE-ABS("narrative*") OR TITLE-ABS("thematic analysis") OR TITLE-ABS("content analysis") OR TITLE-ABS("grounded theory") OR TITLE-ABS("phenomenology*") OR TITLE-ABS("life history") OR TITLE-ABS("participant observation") OR TITLE-ABS("open-ended") OR TITLE-ABS("unstructured interview") OR TITLE-ABS("interpretive") OR TITLE-ABS("lived experience*") OR TITLE-ABS("case studies") OR TITLE-ABS("ethnography*") OR TITLE-ABS("in-depth*") OR TITLE-ABS("semi-structured")) |
| **3** | (India[Mesh] OR India[tiab]) | (India OR India) | ('india'/exp OR india:ti,ab,kw) | (MH "India+") OR (TI India OR AB India) | (INDEXTERMS("India") OR TITLE-ABS("India")) |
|  |  | "unsafe abortion" OR "induced abortion" OR abortion OR "abortion practices" OR "abortion methods" OR "abortion decision" OR "unsafe MTP*" OR miscarriage* OR "unsafe termination of pregnancy*" (Topic) and qualitative OR interview* OR "focus group*" OR exploratory OR experiences OR perception* OR belief* OR barrier* OR narrative* OR "thematic analysis" OR "content analysis" OR "grounded theory" OR phenomenolog* OR "life history" OR "participant observation" OR open-ended OR "unstructured interview*" OR interpretive OR "lived experience*" OR "case stud*" OR ethnograph* OR in-depth* OR semi-structured (Topic) and India OR Indian (Topic) |  |  |  |
|  | #1 AND #2 AND #3 | #1 AND #2 AND #3 | #1 AND #2 AND #3 | #1 AND #2 AND #3 | #1 AND #2 AND #3 |
| **4** | ("unsafe abortion"[Mesh] OR "unsafe abortion"[tiab] OR "abortion, induced"[Mesh] OR abortion[tiab] OR "abortion practices"[tiab] OR "abortion methods"[tiab] OR "abortion decision"[tiab]) OR "unsafe mtp"[tiab] OR miscarriage[tiab] OR "unsafe termination of pregnancy"[tiab] AND ("qualitative research"[Mesh] OR qualitative[tiab] OR interview[tiab] OR "focus group"[tiab] OR exploratory[tiab] OR experiences[tiab] OR perception*[tiab] OR beliefs[tiab] OR barriers[tiab] OR narrative*[tiab] OR "thematic analysis"[tiab] OR "content analysis"[tiab] OR "grounded theory"[tiab] OR  phenomenology*[tiab] OR "life history"[tiab] OR "participant observation"[tiab] OR open-ended[tiab] OR "unstructured interview"[tiab] OR interpretive[tiab] OR "lived experience*"[tiab] OR "case studies"[tiab] OR ethnography*[tiab] OR in-depth*[tiab] OR semi-structured[tiab]) AND (India[Mesh] OR India[tiab]) | ("unsafe abortion" OR "unsafe abortion" OR "abortion, induced" OR abortion OR "abortion practices" OR "abortion methods" OR "abortion decision") OR "unsafe mtp" OR miscarriage OR "unsafe termination of pregnancy" AND  ("qualitative research" OR qualitative OR interview OR "focus group" OR exploratory OR experiences OR perception* OR beliefs OR barriers OR narrative* OR "thematic analysis" OR "content analysis" OR "grounded theory" OR phenomenology* OR "life history" OR "participant observation" OR open-ended OR "unstructured interview" OR interpretive OR "lived experience*" OR "case studies" OR ethnography* OR in-depth* OR semi-structured)  AND (India OR India) | ('unsafe abortion'/exp OR 'unsafe abortion':ti,ab,kw OR 'abortion induced'/exp OR abortion:ti,ab,kw OR 'abortion practices':ti,ab,kw OR 'abortion methods':ti,ab,kw OR 'abortion decision':ti,ab,kw OR 'unsafe mtp*':ti,ab,kw OR miscarriage*:ti,ab,kw OR 'unsafe termination of pregnancy*':ti,ab,kw) AND ('qualitative research'/exp OR qualitative:ti,ab,kw OR  interview*:ti,ab,kw OR 'focus group*':ti,ab,kw OR perception*:ti,ab,kw OR experience*:ti,ab,kw OR 'in-depth':ti,ab,kw OR 'semi-structured':ti,ab,kw OR 'case stud*':ti,ab,kw OR ethnograph*:ti,ab,kw OR 'grounded theory':ti,ab,kw OR phenomenolog*:ti,ab,kw OR 'thematic analysis':ti,ab,kw OR 'content analysis':ti,ab,kw) AND ('india'/exp OR india:ti,ab,kw) | (MH "Abortion, Induced") OR (MH "Abortion, Therapeutic") OR (MH "Abortion, Legal") OR (MH "Abortion, Criminal") OR (MH "Abortion") OR ("unsafe abortion" OR abortion OR "abortion practices" OR "abortion methods" OR "abortion decision" OR "unsafe termination of pregnancy" OR miscarriage*))  AND ((MH "Qualitative Research+") OR (TI qualitative OR AB qualitative) OR (TI interview* OR AB interview*) OR (TI "focus group*" OR AB "focus group*") OR (TI exploratory OR AB exploratory) OR (TI experiences OR AB experiences) OR (TI perception* OR AB perception*) OR (TI beliefs OR AB beliefs) OR (TI barriers OR AB barriers) OR (TI narrative* OR AB narrative*) OR (TI "thematic analysis" OR AB "thematic analysis") OR (TI "content analysis" OR AB "content analysis") OR (TI "grounded theory" OR AB "grounded theory") OR (TI phenomenology* OR AB phenomenology*) OR (TI "life history" OR AB "life history") OR (TI "participant observation" OR AB "participant observation") OR (TI open-ended OR AB open-ended) OR (TI "unstructured interview*" OR AB "unstructured interview*") OR (TI interpretive OR AB interpretive) OR (TI "lived experience*" OR AB "lived experience*") OR (TI "case stud*" OR AB "case stud*") OR (TI ethnography* OR AB ethnography*) OR (TI in-depth* OR AB in-depth*) OR (TI semi-structured OR AB semi-structured)) AND (((MH "India") OR India) | (INDEXTERMS("unsafe abortion") OR TITLE-ABS("unsafe abortion") OR INDEXTERMS("abortion, induced") OR TITLE-ABS("abortion") OR TITLE-ABS("abortion practices") OR TITLE-ABS("abortion methods") OR TITLE-ABS("abortion decision")) OR TITLE-ABS("unsafe mtp") OR TITLE-ABS("miscarriage") OR TITLE-ABS("unsafe termination of pregnancy") AND (INDEXTERMS("qualitative research") OR TITLE-ABS("qualitative") OR TITLE-ABS("interview") OR TITLE-ABS("focus group") OR TITLE-ABS("exploratory") OR TITLE-ABS("experiences") OR TITLE-ABS("perception*") OR TITLE-ABS("beliefs") OR TITLE-ABS("barriers") OR TITLE-ABS("narrative*") OR TITLE-ABS("thematic analysis") OR TITLE-ABS("content analysis") OR TITLE-ABS("grounded theory") OR TITLE-ABS("phenomenology*") OR TITLE-ABS("life history") OR TITLE-ABS("participant observation") OR TITLE-ABS("open-ended") OR TITLE-ABS("unstructured interview") OR TITLE-ABS("interpretive") OR TITLE-ABS("lived experience*") OR TITLE-ABS("case studies") OR TITLE-ABS("ethnography*") OR TITLE-ABS("in-depth*") OR TITLE-ABS("semi-structured")) AND (INDEXTERMS("India") OR TITLE-ABS("India")) |
|  | 205 | 420 | 415 | 172 |  |

GIM (14 March 2026): (("unsafe abortion" OR "induced abortion" OR abortion OR "abortion practices" OR "abortion methods" OR "abortion decision" OR "unsafe MTP*" OR miscarriage* OR "unsafe termination of pregnancy*")) AND ((qualitative OR interview* OR "focus group*" OR exploratory OR experiences OR perception* OR belief* OR barrier* OR narrative* OR "thematic analysis" OR "content analysis" OR "grounded theory" OR phenomenolog* OR "life history" OR "participant observation" OR open-ended OR "unstructured interview*" OR interpretive OR "lived experience*" OR "case stud*" OR ethnograph* OR in-depth* OR semi-structured)) AND ((India OR Indian))

Total hits: 97
