# Supplementary material for: Addressing unsafe abortion in India: A mixed-methods systematic review protocol
Source: PLoS One. 2026 Oct 27;21(10):e0359623. doi: 10.1371/journal.pone.0359623 (PMC13712606; doi:10.1371/journal.pone.0359623)
Supplement: Supplementary file 4 [file pone.0359623.s004.docx]

Dear Editor,

PLOS ONE Journal

Thank you for your email and for providing us the opportunity to address the technical queries regarding our resubmission.

Please find below our point-by-point responses to the issues raised.

Thank you for your time and consideration.

Kind regards,

Dr. Reema Mukherjee (on behalf of all authors)

Corresponding Author

Email: rima.mukerjee@gmail.com

**EDITOR’S COMMENT AND AUTHOR RESPONSES**

## COMMENT 1: We note that this manuscript is a scoping review; our author guidelines require that you upload a PRISMA-ScR checklist as supporting information.

**Response:-** We respectfully clarify that this manuscript describes a **mixed-methods systematic review protocol**, not a scoping review.

Unlike scoping reviews, our protocol includes:

- Formal risk-of-bias assessment using validated tools (Newcastle–Ottawa Scale, RoB 2, JBI/CASP, MMAT)
- Quantitative meta-analysis where appropriate
- Pre-specified primary and secondary outcomes
- Subgroup analyses
- Certainty of evidence assessment using GRADE and GRADE-CERQual

These elements align with established methodological standards for systematic reviews rather than scoping reviews.

Accordingly, the manuscript adheres to the **PRISMA-P (Preferred Reporting Items for Systematic Review and Meta-Analysis Protocols)** guidelines. The PRISMA-P checklist has been uploaded as supporting information.

We kindly request that the manuscript be evaluated under the framework of a systematic review protocol.

**COMMENT 2:** The manuscript appears to be a resubmission of PONE-D-26-01272. Please provide a point-by-point response to the original reviewers’ comments

Response: Thank you for this reminder. We confirm that this submission is a revised version of the previously considered manuscript (PONE-D-26-01272).

A detailed point-by-point rebuttal letter addressing all original reviewer comments has been uploaded with this resubmission. The manuscript has been substantially revised to address the prior concerns, including:

- Removal of all language restrictions
- Strengthening of the search strategy
- Clarification of methodological framework
- Detailed specification of risk-of-bias tools
- Expanded description of quantitative and qualitative synthesis methods
- Clear statement of OSF registration

We believe these revisions comprehensively address the issues raised previously.

**Original Reviewer Comment 1:** Language restrictions are imposed on the literature searches without adequate justification. Exclusion of studies published in other languages can increase the risk of bias in systematic reviews.

Author Response: We fully agree with this concern. In the original submission, we had inadvertently included a language restriction. We have now completely removed all language restrictions from our methodology. The following changes have been made:

| Action | Location in Manuscript |
| --- | --- |
| Deleted the sentence "Only publications written in English or available in an English translation will be reviewed" | Eligibility criteria section (Page 6) |
| Added explicit statement "No language restrictions will be applied to the searches" | Information sources and search strategy section (Page 7) |
| Added strengths statement highlighting this | Discussion section, limitations paragraph (Page 11-12) |

**Original Reviewer Comment 2:** Any restrictions should be justified based on scientific reasons rather than resource constraints.

Author Response: We have removed all restrictions entirely**.**

**Original Reviewer Comment 3:** The manuscript does not meet PLOS ONE standards for systematic reviews.

Author Response: We have substantially strengthened the methodology to meet PLOS ONE standards:

| Area | Improvements Made |
| --- | --- |
| Search Strategy | Clarified that searches will cover major international databases (PubMed/MEDLINE, Embase, Scopus, Web of Science, CINAHL) and Indian databases (IndMED, MedIND, J-Gate) to ensure comprehensive coverage |
| Language | Removed all language restrictions and added explicit statement "No language restrictions will be applied to the searches" to minimize bias |
| Quality Assessment | Clearly specified the use of Newcastle-Ottawa Scale for observational studies, Cochrane RoB 2 for trials, CASP/JBI for qualitative studies, and MMAT for mixed-methods studies |
| Synthesis | Detailed the random-effects meta-analysis approach for quantitative data and thematic synthesis for qualitative data with clear explanation of methods |
| Integration | Explained the convergent-segregated mixed-methods approach with joint displays for integrating quantitative and qualitative findings |
| Limitations | Added a comprehensive Strengths and Limitations section addressing heterogeneity, facility-based bias, and subnational data gaps |
| Registration | Confirmed protocol registration with Open Science Framework (OSF) as required |

**Comment 3:-**

Request for Authorship Change(s) form attached.

Thank you for your consideration.

Sincerely,

Dr. Reema Mukherjee

Scientist-E (Medical)

Division of Reproductive, Child Health, and Nutrition

Indian Council of Medical Research, Delhi

Email: rima.mukerjee@gmail.com
