# Supplementary material for: Addressing unsafe abortion in India: A mixed-methods systematic review protocol
Source: PLoS One. 2026 Oct 27;21(10):e0359623. doi: 10.1371/journal.pone.0359623 (PMC13712606; doi:10.1371/journal.pone.0359623)
Supplement: Supplementary file 5 [file pone.0359623.s005.docx]

**Addressing Unsafe abortion in India: A mixed-methods systematic review protocol**

| **Sl. No.** | **Reviewer/Editor Comment** | **Author’s Response** | **Page No.** | **Line No.** |
| --- | --- | --- | --- | --- |
| **Editor comments** | |  |  |  |
| 1 | The manuscript does not fully adhere to PLOS ONE reporting requirements for systematic review protocols. | The manuscript has been revised to improve compliance with the PLOS ONE reporting requirements for systematic review protocols. Specifically, the Methodology section has been revised to clarify that the protocol has been prepared in accordance with the Preferred Reporting Items for Systematic Review and Meta-Analysis Protocols (PRISMA-P) guideline. In addition, the protocol includes the key methodological components recommended by PRISMA-P, including protocol registration, eligibility criteria, information sources, search strategy, study selection, data extraction, outcomes, risk-of-bias assessment, data synthesis, certainty of evidence assessment, and mixed-methods integration | 6 | 139-141 |
| 2 | Although the protocol states that it follows PRISMA-P guidelines, several methodological and reporting components remain insufficiently described. Authors are encouraged to ensure complete compliance with PRISMA-P and other relevant methodological guidance for mixed-methods systematic reviews | The manuscript has been revised to provide additional methodological detail in accordance with PRISMA-P and mixed-methods systematic review guidance. Specifically, we have clarified the procedures for pilot testing of the data extraction form, management of overlapping datasets, and planned sensitivity analyses, thereby strengthening the methodological transparency of the protocol | 8 and 10 | 199-200, 211-213, 267-268 |
| 3 | The rationale for the review requires substantial strengthening. The manuscript does not clearly identify the specific evidence gap, explain how this review differs from existing reviews, or demonstrate its anticipated contribution to the literature, policy, and practice | The rationale has been strengthened by clearly highlighting the existing evidence gap, explaining how the proposed review differs from previous reviews, and clarifying its relevance to policy and practice in the Indian context. | 6 | 121-124 |
| 4 | The background literature appears incomplete. Several important India-specific studies, including analyses based on National Family Health Survey (NFHS) data and other nationally representative datasets, are not adequately discussed. The authors should revisit the literature (<https://doi.org/10.1017/S0021932023000081>; <https://link.springer.com/article/10.1186/s12889-022-13912-4>; <https://doi.org/10.1017/S002193202300024X>; <https://link.springer.com/chapter/10.1007/978-981-95-1806-7_11>; and many more papers are missing) and refine the study rationale accordingly. | The Background section has been revised to include additional India-specific evidence from nationally representative datasets (including NFHS-based studies) to strengthen the study rationale and provide a more comprehensive overview of the existing literature. | 5 | 98-106 |
| 5 | A. The search strategy requires further refinement. The selection of MeSH terms and keywords appears limited and may not capture all relevant evidence. A more comprehensive, reproducible, and peer-reviewed search strategy is recommended. B. Additional methodological details are needed regarding study selection, management of overlapping datasets, assessment of publication bias, sensitivity analyses, and the planned integration of quantitative and qualitative findings | A. We carefully reviewed the search strategy to improve its clarity, transparency, and reproducibility. As the searches had already been completed using the predefined strategy, the executed search strategy was retained to preserve methodological consistency and reproducibility. The complete search strategy has been provided in the Supplementary Material B. Additional methodological details have been incorporated regarding the management of overlapping datasets and planned sensitivity analyses. The protocol already describes the study selection process, publication bias assessment, and integration of quantitative and qualitative findings. | 8  9,10 | 190-191  223-225, 279-280 |
| 6 | The strengths and limitations of the proposed review are only briefly discussed. A more balanced consideration of potential methodological challenges and sources of bias would strengthen the protocol. | Thank you for this valuable comment. We believe that this aspect is already addressed in the Discussion section, which outlines the anticipated strengths of the review as well as key methodological challenges and potential sources of bias, including heterogeneity in definitions, reporting differences, under-reporting due to stigma, and limitations in subnational data availability. | 13 | 354-363 |
|  |  |  |  |  |
| **Reviewer #1** | |  |  |  |
| 7 | Previous data show that unsafe abortion in India causes several complications, due to unsafe methods, or untrained providers. With the MTP amendment act (2021), these problems may reduce. The authors may try to compare publications prior to the MTP amendment act (2021) and after it was in place. This may provide some insight into whether the expected improvement in the womens' outcome after abortion is there or not | We agree that examining evidence before and after the Medical Termination of Pregnancy (Amendment) Act, 2021 may provide useful insights into changes in unsafe abortion outcomes. Accordingly, we have incorporated policy-era subgroup analyses in the protocol, including a post-2021 subgroup, to explore temporal variations in outcomes where sufficient data are available. We acknowledge that the number of studies published after the 2021 amendment may be limited, and therefore any such comparisons will be interpreted with appropriate caution. | 11 | 310-319 |
| **Reviewer #2** | |  |  |  |
| 8 | search strategy is including "miscarriage"[Title/Abstract] will retrieve spontaneous abortions but it is excluded from the study, at 2 places " MeSH term" the gap is omitted, bracket inconsistency, Boolean operators’ correction needed at some places. So, the Search strategy need to be rechecked. | **Thank you for this valuable comment.** The search strategy was designed to maximise the sensitivity of the search and ensure comprehensive retrieval of potentially relevant records. We carefully reviewed the search strategy in light of your comment and retained the broader search terms to minimise the risk of missing studies in which induced abortion may not be consistently indexed or described. Although the inclusion of the term *"miscarriage"* may retrieve studies on spontaneous abortion, studies focusing exclusively on spontaneous abortion will be excluded during title/abstract and full-text screening according to the predefined eligibility criteria. As the searches had already been completed using this strategy, the executed search strategy was retained to preserve methodological consistency and reproducibility. We have, however, carefully reviewed and corrected minor formatting, parentheses, Boolean syntax, quotation marks, spacing, and wildcard notation inconsistencies in the supplementary search strategy to improve transparency and reproducibility without altering the executed search strategy or retrieved record. | 19-20 |  |
| 9 | The reference 24(definition for unsafe abortion) need to be corrected | The definition of unsafe abortion has been revised to align with the terminology used in the WHO *Abortion Care Guideline* (2022), and the corresponding reference has been corrected | 9 | 237-241 |
| 10 | Authors may consider registering the protocol in PROSPERO. | The protocol has been registered with the Open Science Framework (OSF). The OSF registration details have been added to the manuscript. | 7 | 157-159 |
| 11 | Study plans to assess heterogeneity with I² but no thresholds specified for "acceptable" heterogeneity. | The Statistical Analysis section has been revised to specify the interpretation of the I² statistic for assessing statistical heterogeneity, in accordance with the recommendations of the Cochrane Handbook for Systematic Reviews of Interventions. | 10 | 277-280 |
