# Supplementary material for: Addressing unsafe abortion in India: A mixed-methods systematic review protocol
Source: PLoS One. 2026 Oct 27;21(10):e0359623. doi: 10.1371/journal.pone.0359623 (PMC13712606; doi:10.1371/journal.pone.0359623)
Supplement: Peer review history

# Author comment: Author response to Decision Letter 0

**DOI:** 10.1371/journal.pone.0359623.r001

**Transfer Alert**

This paper was transferred from another journal. As a result, its full editorial history (including decision letters, peer reviews and author responses) may not be present.

19 Mar 2026

Attachment Submitted filename: rebutal_unsafe abortion 030326.docx

# Peer review: Decision Letter 0

**DOI:** 10.1371/journal.pone.0359623.r002

**Authors:** Margubur Rahaman

11 Jun 2026

Dear Dr. Mukherjee,

plosone@plos.org. When you're ready to submit your revision, log on to https://www.editorialmanager.com/pone/ and select the 'Submissions Needing Revision' folder to locate your manuscript file.

- A letter that responds to each point raised by the academic editor and reviewer(s). You should upload this letter as a separate file labeled 'Response to Reviewers'.
- A marked-up copy of your manuscript that highlights changes made to the original version. You should upload this as a separate file labeled 'Revised Manuscript with Track Changes'.
- An unmarked version of your revised paper without tracked changes. You should upload this as a separate file labeled 'Manuscript'.

If you would like to make changes to your financial disclosure, please include your updated statement in your cover letter. Guidelines for resubmitting your figure files are available below the reviewer comments at the end of this letter.

If applicable, we recommend that you deposit your laboratory protocols in protocols.io to enhance the reproducibility of your results. Protocols.io assigns your protocol its own identifier (DOI) so that it can be cited independently in the future. For instructions see: https://journals.plos.org/plosone/s/submission-guidelines#loc-laboratory-protocols. Additionally, PLOS ONE offers an option for publishing peer-reviewed Lab Protocol articles, which describe protocols hosted on protocols.io. Read more information on sharing protocols at https://plos.org/protocols?utm_medium=editorial-email&utm_source=authorletters&utm_campaign=protocols.

As the corresponding author, your ORCID iD is verified in the submission system and will appear in the published article. PLOS supports the use of ORCID, and we encourage all coauthors to register for an ORCID iD and use it as well. Please encourage your coauthors to verify their ORCID iD within the submission system before final acceptance, as unverified ORCID iDs will not appear in the published article. *Only*  the individual author can complete the verification step; PLOS staff *cannot*  verify ORCID iDs on behalf of authors.

We look forward to receiving your revised manuscript.

Kind regards,

Margubur Rahaman, Ph.D.

Academic Editor

PLOS One

Journal Requirements:

When submitting your revision, we need you to address these additional requirements.

1. Please ensure that your manuscript meets PLOS ONE's style requirements, including those for file naming. The PLOS ONE style templates can be found at

https://journals.plos.org/plosone/s/file?id=wjVg/PLOSOne_formatting_sample_main_body.pdf and

https://journals.plos.org/plosone/s/file?id=ba62/PLOSOne_formatting_sample_title_authors_affiliations.pdf.

2. Thank you for stating in your Funding Statement:

“This work was supported by the Indian Council of Medical Research (ICMR), Government of India, through intramural research funding (Grant No.: 5/7/RCN/Intramural/28.07.2025). The funder had no role in the study design, data extraction, analysis, interpretation of findings, or preparation of this manuscript.”

Please provide an amended statement that declares *all* the funding or sources of support (whether external or internal to your organization) received during this study, as detailed online in our guide for authors at http://journals.plos.org/plosone/s/submit-now.  Please also include the statement “There was no additional external funding received for this study.” in your updated Funding Statement.

Please include your amended Funding Statement within your cover letter. We will change the online submission form on your behalf.

3. When completing the data availability statement of the submission form, you indicated that you will make your data available on acceptance. We strongly recommend all authors decide on a data sharing plan before acceptance, as the process can be lengthy and hold up publication timelines. Please note that, though access restrictions are acceptable now, your entire data will need to be made freely accessible if your manuscript is accepted for publication. This policy applies to all data except where public deposition would breach compliance with the protocol approved by your research ethics board. If you are unable to adhere to our open data policy, please kindly revise your statement to explain your reasoning and we will seek the editor's input on an exemption. Please be assured that, once you have provided your new statement, the assessment of your exemption will not hold up the peer review process.

4. Thank you for stating the following in your manuscript:

“This work is supported by the Regional Medical Research Centre (RMRC), Bhubaneswar, Indian Council of Medical Research (ICMR). The funder had no role in study design, data collection, analysis, interpretation, or manuscript preparation.”

We note that you have provided additional information within the Acknowledgements Section that is not currently declared in your Funding Statement. Please note that funding information should not appear in the Acknowledgments section or other areas of your manuscript. We will only publish funding information present in the Funding Statement section of the online submission form.

Please remove any funding-related text from the manuscript and let us know how you would like to update your Funding Statement. Currently, your Funding Statement reads as follows:

“This work was supported by the Indian Council of Medical Research (ICMR), Government of India, through intramural research funding (Grant No.: 5/7/RCN/Intramural/28.07.2025). The funder had no role in the study design, data extraction, analysis, interpretation of findings, or preparation of this manuscript.”

Please include your amended statements within your cover letter; we will change the online submission form on your behalf.

5. PLOS requires an ORCID iD for the corresponding author in Editorial Manager on papers submitted after December 6th, 2016. Please ensure that you have an ORCID iD and that it is validated in Editorial Manager. To do this, go to ‘Update my Information’ (in the upper left-hand corner of the main menu), and click on the Fetch/Validate link next to the ORCID field. This will take you to the ORCID site and allow you to create a new iD or authenticate a pre-existing iD in Editorial Manager.

6.We notice that your supplementary tables are included in the manuscript file. Please remove them and upload them with the file type 'Supporting Information'. Please ensure that each Supporting Information file has a legend listed in the manuscript after the references list.

7. Please include captions for your Supporting Information files at the end of your manuscript, and update any in-text citations to match accordingly. Please see our Supporting Information guidelines for more information: http://journals.plos.org/plosone/s/supporting-information.

8. If the reviewer comments include a recommendation to cite specific previously published works, please review and evaluate these publications to determine whether they are relevant and should be cited. There is no requirement to cite these works unless the editor has indicated otherwise.

Additional Editor Comments:

Thank you for submitting your protocol manuscript. The topic is important and highly relevant to reproductive health research in India. However, several issues require attention before the manuscript can be considered further.

1. The manuscript does not fully adhere to PLOS ONE reporting requirements for systematic review protocols. Please carefully review the PLOS ONE Submission Guidelines and ensure that all required reporting elements are adequately addressed.

2. Although the protocol states that it follows PRISMA-P guidelines, several methodological and reporting components remain insufficiently described. Authors are encouraged to ensure complete compliance with PRISMA-P and other relevant methodological guidance for mixed-methods systematic reviews.

3. The rationale for the review requires substantial strengthening. The manuscript does not clearly identify the specific evidence gap, explain how this review differs from existing reviews, or demonstrate its anticipated contribution to the literature, policy, and practice.

4. The background literature appears incomplete. Several important India-specific studies, including analyses based on National Family Health Survey (NFHS) data and other nationally representative datasets, are not adequately discussed. The authors should revisit the literature (https://doi.org/10.1017/S0021932023000081; https://link.springer.com/article/10.1186/s12889-022-13912-4; https://doi.org/10.1017/S002193202300024X; https://link.springer.com/chapter/10.1007/978-981-95-1806-7_11; and many more papers are missing) and refine the study rationale accordingly.

5. The search strategy requires further refinement. The selection of MeSH terms and keywords appears limited and may not capture all relevant evidence. A more comprehensive, reproducible, and peer-reviewed search strategy is recommended.

Additional methodological details are needed regarding study selection, management of overlapping datasets, assessment of publication bias, sensitivity analyses, and the planned integration of quantitative and qualitative findings.

6. The strengths and limitations of the proposed review are only briefly discussed. A more balanced consideration of potential methodological challenges and sources of bias would strengthen the protocol.

7. Consider incorporating visual representations of the evidence base in the final review. Mapping included studies across time (publication period), space (states/regions), and population groups would improve readability, facilitate identification of evidence gaps, and enhance the policy relevance of the review.

I encourage the authors to substantially revise the manuscript in line with established reporting and methodological standards before resubmission.

Reviewers' comments:

Reviewer's Responses to Questions

**Comments to the Author**

1. Is the manuscript technically sound, and do the data support the conclusions?

Reviewer #1: Yes

Reviewer #2: Yes

**********

2. Has the statistical analysis been performed appropriately and rigorously?

Reviewer #1: Yes

Reviewer #2: I Don't Know

**********

3. Have the authors made all data underlying the findings in their manuscript fully available?

The PLOS Data policy

Reviewer #1: Yes

Reviewer #2: Yes

**********

4. Is the manuscript presented in an intelligible fashion and written in standard English?

Reviewer #1: Yes

Reviewer #2: Yes

**********

Reviewer #1: Previous data show that unsafe abortion in India causes several complications, due to unsafe methods, or untrained providers. With the MTP amendment act (2021), these problems may reduce. The authors may try to compare publications prior to the MTP amendment act (2021) and after it was in place. This may provide some insight into whether the expected improvement in the womens' outcome after abortion is there or not.

Reviewer #2: search strategy is including "miscarriage"[Title/Abstract] will retrieve spontaneous abortions but it is excluded from the study, at 2 places " MeSH term" the gap is omitted, bracket inconsistency , Boolean operators correction needed at some places. So the Search strategy need to be rechecked. The reference 24(defination for unsafe abortion ) need to be corrected. If authors want they can resister in PROSPERO aslo. study plans to assess heterogeneity with I² but no thresholds specified for "acceptable" heterogeneity

**********

what does this mean?). If published, this will include your full peer review and any attached files.

If you choose “no”, your identity will remain anonymous but your review may still be made public.

**Do you want your identity to be public for this peer review?**  For information about this choice, including consent withdrawal, please see our Privacy Policy

Reviewer #1: **Yes:**  Dr Rashmi Bagga

Reviewer #2: No

**********

[NOTE: If reviewer comments were submitted as an attachment file, they will be attached to this email and accessible via the submission site. Please log into your account, locate the manuscript record, and check for the action link "View Attachments". If this link does not appear, there are no attachment files.]

To ensure your figures meet our technical requirements, please review our figure guidelines: https://journals.plos.org/plosone/s/figures

You may also use PLOS’s free figure tool, NAAS, to help you prepare publication quality figures: https://journals.plos.org/plosone/s/figures#loc-tools-for-figure-preparation.

NAAS will assess whether your figures meet our technical requirements by comparing each figure against our figure specifications.

# Author comment: Author response to Decision Letter 1

**DOI:** 10.1371/journal.pone.0359623.r003

20 Jul 2026

We thank the Academic Editor and reviewers for their constructive comments. We have addressed all comments and revised the manuscript accordingly. A detailed point-by-point response is provided in the uploaded "Response to Reviewers" document, and all revisions are highlighted in the tracked-changes manuscript.

Attachment Submitted filename: Response to Reviewers.docx

# Editor report: Decision Letter 1

**DOI:** 10.1371/journal.pone.0359623.r004

**Authors:** Margubur Rahaman

16 Sep 2026

Addressing Unsafe abortion in India: A mixed-methods systematic review protocol

PONE-D-26-08307R1

Dear Dr. Mukherjee,

We’re pleased to inform you that your manuscript has been judged scientifically suitable for publication and will be formally accepted for publication once it meets all outstanding technical requirements.

Within one week, you’ll receive an e-mail detailing the required amendments. When these have been addressed, you’ll receive a formal acceptance letter and your manuscript will be scheduled for publication.

An invoice will be generated when your article is formally accepted. Please note, if your institution has a publishing partnership with PLOS and your article meets the relevant criteria, all or part of your publication costs will be covered. Please make sure your user information is up-to-date by logging into Editorial Manager at Editorial Manager® and clicking the ‘Update My Information' link at the top of the page. For questions related to billing, please contact billing support.

If your institution or institutions have a press office, please notify them about your upcoming paper to help maximize its impact. If they’ll be preparing press materials, please inform our press team as soon as possible -- no later than 48 hours after receiving the formal acceptance. Your manuscript will remain under strict press embargo until 2 pm Eastern Time on the date of publication. For more information, please contact onepress@plos.org.

Kind regards,

Margubur Rahaman, Ph.D.

Academic Editor

PLOS One

Additional Editor Comments (optional):

Reviewers' comments:

# Editor report: Acceptance letter

**DOI:** 10.1371/journal.pone.0359623.r005

**Authors:** Margubur Rahaman

PONE-D-26-08307R1

PLOS One

Dear Dr. Mukherjee,

I'm pleased to inform you that your manuscript has been deemed suitable for publication in PLOS One. Congratulations! Your manuscript is now being handed over to our production team.

At this stage, our production department will prepare your paper for publication. This includes ensuring the following:

* All references, tables, and figures are properly cited

* All relevant supporting information is included in the manuscript submission,

* There are no issues that prevent the paper from being properly typeset

You will receive further instructions from the production team, including instructions on how to review your proof when it is ready. Please keep in mind that we are working through a large volume of accepted articles, so please give us a few days to review your paper and let you know the next and final steps.

Lastly, if your institution or institutions have a press office, please let them know about your upcoming paper now to help maximize its impact. If they'll be preparing press materials, please inform our press team within the next 48 hours. Your manuscript will remain under strict press embargo until 2 pm Eastern Time on the date of publication. For more information, please contact onepress@plos.org.

You will receive an invoice from PLOS for your publication fee after your manuscript has reached the completed accept phase. If you receive an email requesting payment before acceptance or for any other service, this may be a phishing scheme. Learn how to identify phishing emails and protect your accounts at https://explore.plos.org/phishing.

If we can help with anything else, please email us at customercare@plos.org.

Thank you for submitting your work to PLOS One and supporting open access.

Kind regards,

PLOS One Editorial Office Staff

on behalf of

Dr. Margubur Rahaman

Academic Editor

PLOS One
